# Investigation of risk signatures associated with anoikis in thyroid cancer through integrated transcriptome and Mendelian randomization analysis

**DOI:** 10.3389/fendo.2024.1458956

**Published:** 2024-11-06

**Authors:** Xiang-Yi Chen, Jia-Ying Lai, Wen-Jun Shen, Dawei Wang, Zhi-Xiao Wei

**Affiliations:** ^1^ Department of Nuclear Medicine, The First Affiliated Hospital of Guangxi Medical University, Nanning, China; ^2^ Department of Medical Engineering, Medical Supplies Center of PLA General Hospital, Beijing, China; ^3^ Department of Nuclear Medicine, The Sixth Medical Center of PLA General Hospital, Beijing, China

**Keywords:** thyroid cancer, anoikis, risk signature, prognosis, Mendelian randomization analysis

## Abstract

**Background:**

Anoikis is intricately associated with the malignant progression of cancer. Thyroid cancer (THCA) is the most common endocrine tumor, metastasis is closely related to treatment response and prognosis of THCA. Hence, it is imperative to comprehensively identify predictive prognostic genes and novel molecular targets for effective THCA therapy.

**Methods:**

Differential expression analysis and weighted gene co-expression network analysis (WGCNA) were utilized to mine differentially expressed anoikis-related (DE-ARGs). Then, the prognostic genes were identified and a risk signature was constructed for THCA using univariate Cox analysis and least absolute shrinkage and selection operator (LASSO) method. Furthermore, the associations between risk signature and immune infiltration, immunotherapy, as well as potential mechanisms of action were determined using multiple R packages and Wilcoxon test. Finally, Mendelian randomized (MR) analysis was conducted to investigate the causal relationship between the prognostic genes and THCA.

**Results:**

In total, six prognostic genes (LRRC75A, METTL7B, ADRA1B, TPD52L1, TNFRSF10C, and CXCL8) related to anoikis were identified, and the corresponding risk signature were constructed to assess the survival time of THCA patients. Immunocorrelation analysis demonstrated the anoikis-relevant risk signature could be used to evaluate immunotherapy effects in THCA patients, and the infiltration of immune cells was correlated with the degree of risk in THCA patients. According to two-sample MR analysis, there was the significant causal relationship between CXCL8 and THCA (odds ratio [OR] > 1 & p< 0.05), and the increase of its gene expression would lead to an increased risk of THCA. Furthermore, real-time quantitative polymerase chain reaction (RT-qPCR) confirmed the upregulated expression patterns of these prognostic genes in THCA tissues.

**Conclusion:**

In conclusion, we constructed the risk signature related to anoikis for THCA, which might have important clinical significance for improving the quality of life and treatment effect of THCA patients.

## 1 Introduction

Thyroid cancer (THCA) is a prevalent malignancy of the endocrine system and ranks as the ninth most common cancer worldwide (1). THCA, primarily originating from follicular cells, constitutes approximately 95% of THCA (2). The prognosis of THCA patients depends on factors such as tumor size, age, degree of metastasis, among which tumor size closely correlates with poor prognosis (3). In the United States alone, around 44,000 new cases of THCA are diagnosed annually. Among patients with highly differentiated THCA, about 54% have a low risk of recurrence and can be effectively treated through surgery (1). Despite having a favorable prognosis overall, once it progresses to locally advanced stages or metastatic radioiodine refractory (RAIR) which exhibits relatively poorer outcomes that significantly impact patient survival rates. Therefore, we believe that identifying novel genes associated with THCA not only plays a crucial role in pathological diagnosis but also provides valuable insights for treatment selection.

Anoikis is a form of cellular apoptosis triggered by the loss or improper attachment (4). Normal epithelial cells rely on extracellular matrix (ECM) contact for survival and proliferation, whereas cancer cells can bypass this requirement (4). Consequently, anti-anoikis mechanisms are essential for invasive metastasis and dissemination of cancer cells (4). Simultaneously, numerous studies have unveiled the association between anoikis and THCA. For instance, Lee JJ et al. discovered that galactin-3 inhibitors suppressed resistance to anoikis and invasion in THCA cells (5). Jensen K et al. demonstrated that inhibiting the gap junction pathway could enhance sensitivity to anoikis stimuli in THCA cells (6). Tang M et al. also identified anoikis-related gene CDKN2A as a predictor of prognosis and immune response while mediating proliferation and migration in THCA (7). Other investigations have revealed that MicroRNA-363-3p inhibits anoikis in human THCA by targeting integrin α6 (8), suggesting a close relationship between anoikis and malignant progression of THCA. Therefore, it is crucial to authenticate predictive genes for effective therapy against THCA.

In order to further investigate the role of anoikis-related genes (ARGs) in the occurrence and development of THCA, we utilized sample information from the TCGA dataset to conduct a comprehensive analysis on survival-associated genes. Through bioinformatics analysis, we successfully identified six prognostic genes for THCA, namely LRRC75A, METTL7B, ADRA1B, TPD52L1, TNFRSF10C, and CXCL8. Additionally, the risk signature was constructed to accurately predict patients’ survival time. To elucidate the causal relationship between these prognostic genes and THCA risk, a two-sample Mendelian randomization (MR) analysis was performed using genome-wide association studies (GWAS) data from the OpenGWAS database. The results revealed a significant causal association between CXCL8 and THCA. Collectively, our findings provide a solid foundation for future research on THCA and offer new insights into personalized treatment strategies.

## 2 Materials and methods

### 2.1 Data acquisition

A total of 497 cases of THCA and 56 control cases were gathered from the UCSC Xena database (https://xenabrowser.net/datapages/). Among these, 496 THCA samples were accompanied by survival information and comprehensive clinical characteristics, encompassing age, M/N/T stage, and stage, etc. A set of 338 ARGs were acquired from the published literature (9). Additionally, the GWAS data for THCA and eQTL GWAS data for ARGs in MR analysis were all collected from the OpenGWAS database (https://gwas.mrcieu.ac.uk/). The THCA’s GWAS dataset, namely ebi-a-GCST90018929, consisted of 1,054 cases with THCA and 490,920 control cases (European), encompassing a total of 24,198,226 single-nucleotide polymorphisms (SNPs).

### 2.2 Differential expression analysis and construction of the WGCNA network

“DESeq2” package (v1.36.0) (10) was utilized to mine the differentially expressed genes (DEGs) between THCA and control cases in the TCGA-THCA dataset with log_2_fold change (FC) > 1 and adj.p< 0.05. To identify key module genes associated with ARGs in THCA, the ssGSEA algorithm in the “GSVA” package (v1.44.5) (11) was utilized to calculate ARG scores for THCA and control specimens in the TCGA-THCA dataset. Subsequently, depending on the gene expression matrix of all samples within the TCGA-THCA dataset and ARG score (clinical trait), a co-expression network was established using “WGCNA” package (v1.70-3) (12). The samples in the TCGA-THCA dataset were subjected to clustering analysis in order to identify and exclude any outlier samples, thereby ensuring the accuracy of subsequent analyses. Afterthat, in order to maximize the adherence of gene interactions to a scale-free distribution, we employed the expression matrix encompassing all genes as input data and conducted computations to determine the optimal soft threshold. By calculating gene adjacency and deriving a coefficient of dissimilarity based on gene similarity, a systematic clustering tree of genes was acquired. Finally, all samples were treated as individual traits and subjected to Pearson correlation analysis with modules obtained from WGCNA analysis in order to identify the module gene exhibiting the highest correlation. The intersection of DEGs and key module genes was performed using the “ggvenn” package (v0.1.9) for visualization, resulting in a set of differentially expressed ARGs (DE-ARGs).

### 2.3 Functional enrichment analysis for DE-ARGs

The protein-protein interaction (PPI) network of DE-ARGs was established through the STRING database (https://www.string-db.org/). Subsequently, “clusterProfiler” package (v4.7.1.001) (13) was employed to perform GO and KEGG enrichment analyses on the DE-ARGs, aiming to mine shared functions and associated pathways among genes.

### 2.4 Screening of prognostic genes and construction of the risk signature

Subsequent analysis involved performing univariate Cox regression using “survival” package (v3.4-0) (14) on DE-ARGs within THCA samples from TCGA-THCA dataset. Genes that met the criteria of hazard ratio (HR) ≠ 1 and p< 0.05 were categorized as survival-associated genes. These genes then underwent proportional hazards (PH) assumption testing. The genes that met the PH assumption (p > 0.05) were further integrated into a LASSO regression analysis using the “glmnet” package (v4.1-6) (15). Optimal model fit was achieved when the lambda value was minimized, identifying the prognostic genes for this study. The THCA samples in TCGA-THCA dataset were randomly allocated to the training set and the internal validation set at a ratio of 7:3 for constructing the risk signature. Based on the prognostic genes and coefficients obtained from the LASSO analysis, the risk score for each THCA sample was calculated according to the formula below:


$$\text{Risk score}={\text{Coefficient}}_{1}*{\text{Gene}}_{1}+{\text{Coefficient}}_{2}*{\text{Gene}}_{2}+\ldots +{\text{Coefficient}}_{n}*{\text{Gene}}_{n}$$

Following the calculation of risk scores for each THCA patient, they were categorized into high-risk and low-risk groups *via* the median risk score. Subsequently, the Kaplan-Meier (K-M) curves for survival analysis between these two risk groups were plotted using “survminer” (v0.4.9) (14) package, followed by a comparison of survival differences through log-rank test. Besides, the receiver operating characteristic (ROC) curves for the risk signature were drawn by “survivalROC” package (v1.0.3) (16), with survival times of 1, 3, and 5 years as the time points. To further assess the generality of the risk signature, we validated it using the same method as described above in the internal validation set.

### 2.5 Independent prognostic analysis

The nomogram was generated using the “rms” package (v6.3-0) (17), and then calibration curves were built to estimate the accuracy of nomograms in predicting 1/3/5-year survival in THCA patients. In order to assess the relevant between prognostic genes and clinical characteristics, we integrated the clinical information (including age, T/M/N stage, and disease stage) of THCA patients obtained from the TCGA database to examine the differences in prognostic gene expression among different clinical features.

### 2.6 Immune infiltration analysis

The infiltration of immune cell types in patients with THCA was estimated with CIBERSORT (https://cibersort.stanford.edu/), and the Wilcoxon test was employed to make comparisons between the high- and low-risk groups. To investigate the association between tumor immune evasion and risk score, we calculated and compared the TIDE scores of THCA samples through TIDE’s official website (http://tide.dfci.harvard.edu/) and Wilcoxon test, respectively. In addition, Spearman correlation analysis was employed to examine the relationship between TIDE and risk scores.

### 2.7 Functional enrichment analysis for prognostic genes

Using “h.all.v7.4.symbols.gmt” obtained from the MSigDB database (http://www.broadinstitute.org/msigdb) as the reference, we employed “GSVA” package (v1.44.5) (11) to calculate scores for various pathways enriched in high- and low-risk groups (p< 0.05). Subsequently, we utilized the “limma” package to identify significantly different pathways between THCA samples in two risk groups. Moreover, the “psych” package (v2.2.9) (18) was utilized for the analysis of Spearman correlation between the risk score and all genes. Depending on risk score and coefficient serving as ranking criteria, gene set enrichment analysis (GSEA) was conducted by the “clusterProfiler” package (|NES| > 1 & adj.p< 0.05). The “c2.cp.kegg.v2023.1.Hs.symbols” gene set obtained from the GSEA website (http://www.gsea-MSigdb.org/gsea/MSigdb) was served as the background gene set.

### 2.8 Drug sensitivity analysis

The oncoPredict package (v0.2) (19) was utilized to calculate the IC50 values of 198 chemotherapeutic/targeted therapy drugs provided by GDSC database (https://www.cancerrxgene.org/) on THCA samples. Subsequently, we conducted a comparative analysis of the IC50 values for 198 drugs between two distinct groups.

### 2.9 Study design of MR analysis

Using a two-sample MR analysis, we examined the causal relationship between prognostic genes and THCA. Therefore, the selection of effective instrumental variables (IVs) should adhere to three crucial assumptions: (1) IVs must exhibit strong associations with exposure; (2) IVs must be independent of confounding factors; and (3) The association between IVs and outcome should be solely mediated by exposure factors.

### 2.10 MR analysis of THCA and prognostic genes

The extract_instruments function in the TwoSampleMR package (v0.5.7) (20) was utilized to identify IVs (SNPs) that exhibited significant correlation with prognostic genes (exposure factors), employing a screening criterion of p< 5*10^-8^. Subsequently, the clump parameter was set to TRUE, and IVs in linkage disequilibrium (LD) were removed by adjusting the parameters as follows: r^2^ = 0.001; kb = 100. To address potential issues arising from IVs exhibiting significant correlation with THCA (outcome), we employed the harmonise_data function in the TwoSampleMR package to standardize effect equiele and effect size. MR analysis was performed using 5 algorithms (MR egger, weighted median, inverse variance weighted [IVW], simple mode, and weighted mode). Notably, the primary outcome considered was based on IVW results, where a *P*< 0.05 indicated a significant causal relationship. Finally, ensuring accuracy in directionality was crucial and achieved through utilizing the TwoSampleMR package to conduct Steiger directivity analysis.

### 2.11 Sensitivity analysis

A sensitivity analysis was undertaken to assure the robustness of MR analysis. Initially, heterogeneity was evaluated using Cochran’s Q statistic and its corresponding p value. Then, the MR egger intercept was examined to investigate potential horizontal pleiotropy. It is worth noting that if p< 0.05 in egger intercept, it necessitates reconsideration of the study design. Furthermore, to demonstrate reliability in our analysis results, a stepwise elimination process was employed where each SNP was gradually removed and its impact on outcome variables assessed. This leave-one-out sensitivity analysis approach served as an additional validation for our findings.

### 2.12 THCA patients and tissue specimens

A total of 15 pairs of THCA tumor and corresponding adjacent normal tissues were collected from First Affiliated Hospital of Guangxi Medical University between August 2024 and September 2024. Samples were snap-frozen and stored at −80°C until used in real-time qPCR (RT-qPCR) experiments. The Research Ethics Committee of The First Affiliated Hospital of Guangxi Medical University approved this study, which was consistent with the Declaration of Helsinki.

### 2.13 RNA Isolation, cDNA Synthesis, and RT-qPCR

Total RNAs were isolated by Triquick Reagent (Trizol Substitute, Solarbio, China). RNA (1 μg), quantified by NanoDrop2000 (Thermo Fisher Scientific), was reversely transcribed to cDNA using the first-strand cDNA synthesis kit (Monad, China). Quantitative PCR was applied using the SYBR Green dye (Yeasen, China) on Real-Time PCR System (Agilen, United States). All primers were synthesized by Sangon Biotech and their sequences were listed in **Supplementary Table S1**. ACTB served as an internal control. The 2^-ΔΔCt^ method was used to determine the relative expression between cancer and normal tissue for each selected prognostic gene.

### 2.14 Statistical analysis

This study was statistically analyzed using the R software package (v4.2.1). Various variables, including expression quantity and infiltration ratio, were evaluated for differences between groups using either the Wilcoxon test or t-test. The significance of discrepancies between groups was typically defined by a p value or adj.p value below 0.05, unless otherwise specified.

## 3 Results

### 3.1 Identification of DE-ARGs in the TCGA-THCA dataset

**Figure 1:**
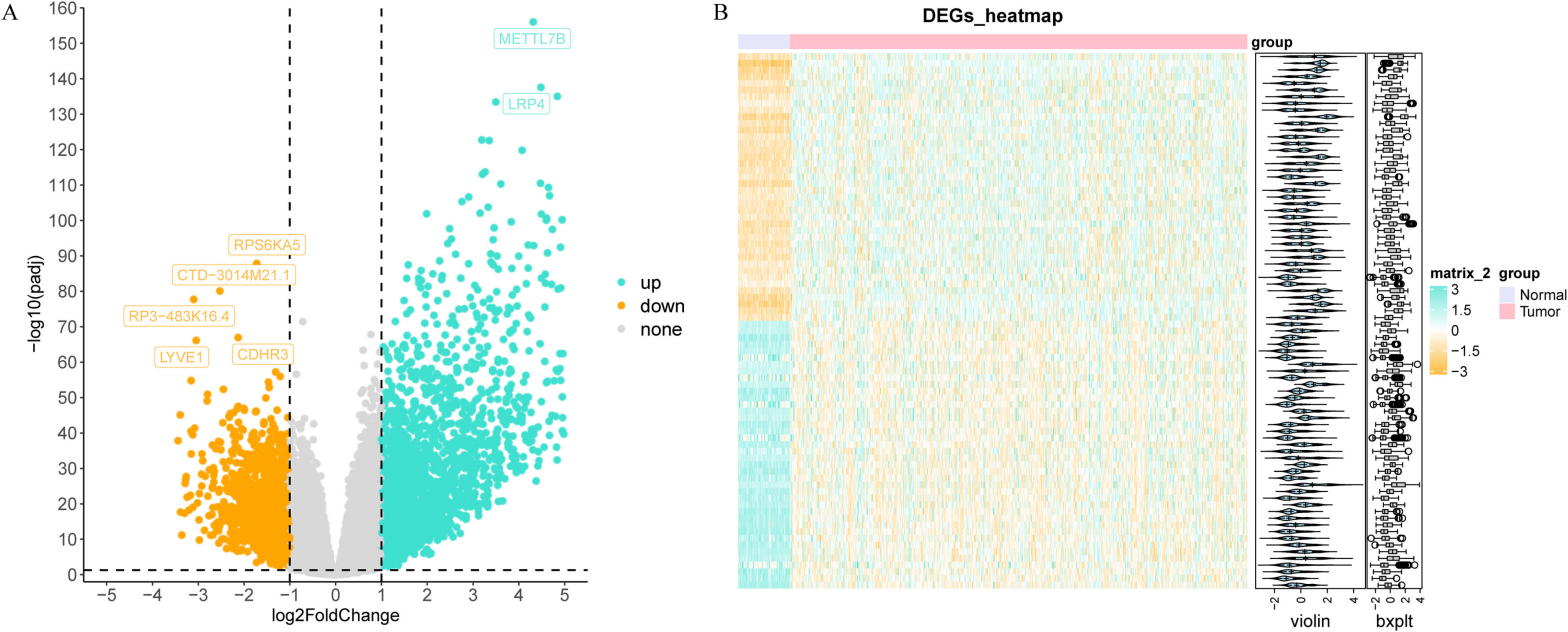
Differential expression analysis in the TCGA-THCA dataset. The volcano map **(A)** and heat map **(B)** of differentially expressed genes (DEGs) between thyroid cancer (THCA) and control groups.

**Figure 2:**
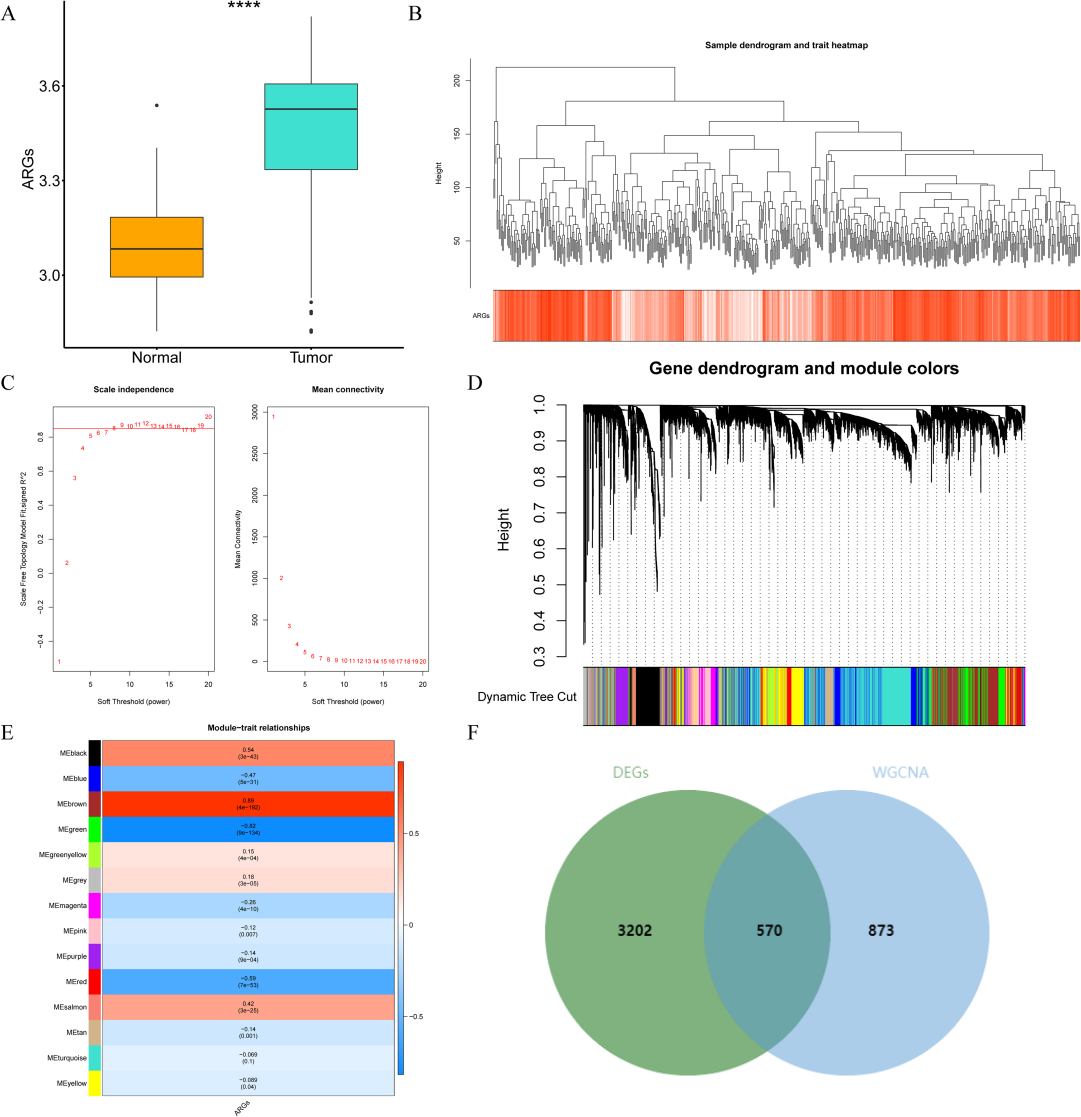
Identification of the differentially expressed anoikis-related genes (DE-ARGs) by constructing the WGCNA network. **(A)** Discrepancies in ARG scores between THCA and control samples. ****p<0.0001. **(B)** The cluster diagram of all samples in the TCGA-THCA dataset. **(C)** Selection of the optimal soft threshold (power). **(D)** Genes were divided into different modules through hierarchical clustering. Different colors represent different modules. **(E)** Correlation heat map between module genes and ARGs. **(F)** The venn diagram of 570 DE-ARGs.

Through differential expression analysis, a set of 3,772 DEGs were detected in the TCGA-THCA cohort, comprising 1,611 down-regulated and 2,161 up-regulated genes. The volcano plot displayed all DEGs, while the heat map depicted the top 80 genes with the most significant up-regulation and down-regulation between control and THCA samples (**Figures 1A, B**, **Supplementary Table S2**). The ARG scores in THCA samples and healthy control samples were compared using the Wilcoxon test, revealing statistically significant differences (p< 0.05, **Figure 2A**). To screen out genes with high ARG correlation, we constructed a WGCNA network based on ARG scores. Depending on the results of the cluster analysis, no outlier samples were identified in the TCGA-THCA dataset, indicating its suitability for conducting WGCNA analysis (**Figure 2B**). When the R2 value approached the threshold of 0.85 (red line) and the average connectivity in the graph on the right tends towards 0, it indicated that the network was approaching a scale-free distribution (**Figure 2C**). Consequently, we determined the optimal soft threshold (β) to be 8. Furthermore, the minimum number of genes was set to 100 according to the standard of the hybrid dynamic tree cutting algorithm, resulting in a total of 14 modules obtained (**Figure 2D**). Among the 14 modules, MEbrown exhibited the strongest association with ARGs score (cor = 0.89 & p< 0.001) (**Figure 2E**). Consequently, MEbrown module was identified as the pivotal module in this study, encompassing a total of 1,443 genes and designated as the key module gene. Finally, 570 DE-ARGs were acquired by overlapping 3,772 DEGs and 1,443 ARGs-related genes (**Figure 2F**).

### 3.2 Investigation of potential action mechanism for DE-ARGs

**Figure 3:**
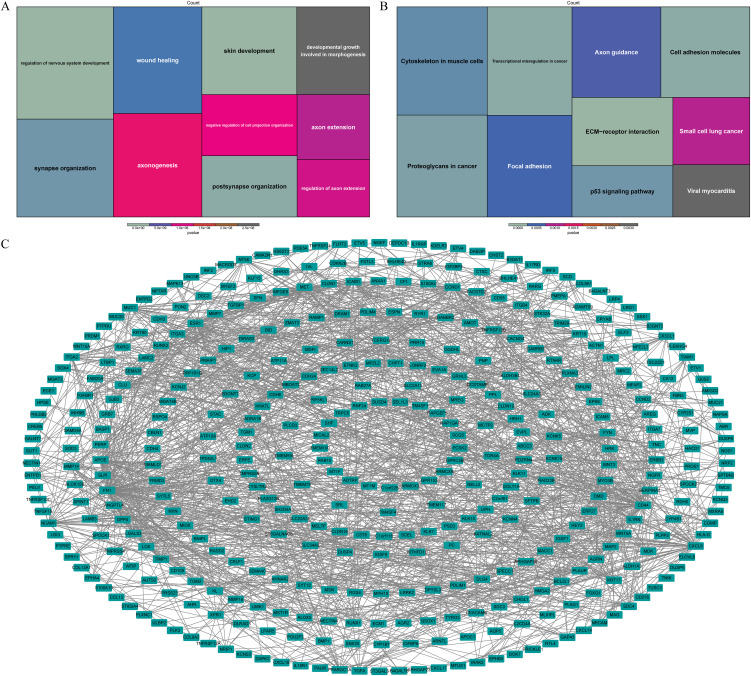
Functional enrichment analysis of DE-ARGs. The GO terms **(A)** and KEGG pathways **(B)** notably enriched by DE-ARGs. **(C)** The PPI network of DE-ARGs.

Based on the functional enrichment analysis, wound healing, developmental growth involved in morphogenesis and pathways associated with nervous system function, such as axon extension, synapse organization, axonogenesis, were enriched by DE-ARGs (**Figure 3A**). Moreover, they were also associated with ECM-receptor interaction, transcriptional misregulation in cancer, cell adhesion molecules, p53 signaling pathway according to KEGG pathways (**Figure 3B**). In order to investigate potential interactions among DE-ARGs, we constructed a PPI network. The network comprised a total of 405 nodes and 1,371 interaction pairs, revealing a highly intricate interplay between 570 ARGs (**Figure 3C**).

### 3.3 Risk signature based on six prognostic genes for THCA

**Figure 4:**
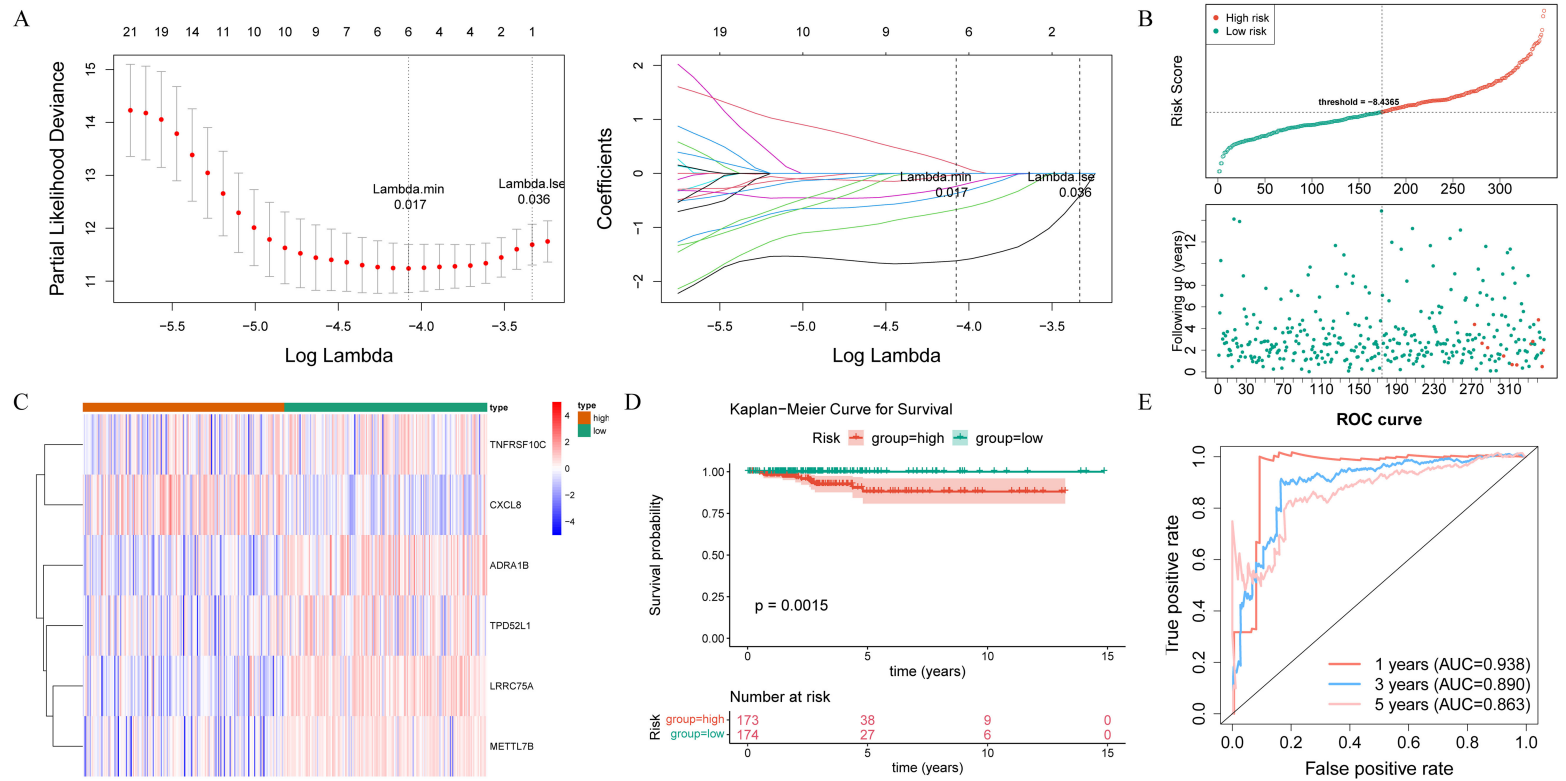
Identification of prognostic genes and construction of the risk signature. **(A)** Identification of optimal log(Lambda) in the LASSO regression analysis, along with their corresponding genes and coefficients, as well as the proportion of residuals explained by the model. **(B)** Risk score distribution, patient survival time, and status. **(C)** The heat map illustrates the expression of prognostic genes. **(D)** Kaplan-Meier (K-M) survival curves for high- and low-risk groups. **(E)** Receiver operating characteristic (ROC) curves showing the predictive efficiency of risk scores. AUC, area under the curve.

After performing univariate Cox regression analysis, a total of 40 survival-associated genes were identified (HR ≠ 1 and p< 0.05) (**Supplementary Table S3**). These genes were subsequently subjected to tests for the PH assumption, which they all satisfied (p > 0.05) (**Supplementary Table S4**). According to LASSO regression analysis, six prognostic genes (LRRC75A, METTL7B, ADRA1B, TPD52L1, TNFRSF10C, and CXCL8) were used to construct the risk signature with the best performance (**Figure 4A**). The THCA samples in the training set were ranked based on their risk scores, and a risk curve was plotted (**Figure 4B**). This curve demonstrated a higher number of deceased samples in the high-risk group compared to the low-risk group, indicating that the mortality rate was lower among low-risk THCA patients. Additionally, **Figure 4C** displayed expression levels of prognostic genes in both high and low-risk groups, in which LRRC75A, METTL7B, ADRA1B, TPD52L1, and TNFRSF10C were low expressed in high-risk group, and CXCL8 showed an opposite trend. The K-M curves revealed a statistically significant disparity in the survival rates of patients with THCA between the high- and low-risk groups (p< 0.05), with the high-risk group exhibiting a higher mortality rate (**Figure 4D**). The area under the curve (AUC) values of the ROC curve were 0.938, 0.890, and 0.863 at 1, 3, and 5 years, respectively, indicating a strong ability for predicting survival time (**Figure 4E**). Furthermore, the risk signature constructed by the prognostic gene in the internal validation set exhibited consistent findings with those observed in the training set, thereby providing further evidence of its validity and universality (**Supplementary Figure S1A–D**).

### 3.4 Risk score was an independent predictor of THCA

**Figure 5:**
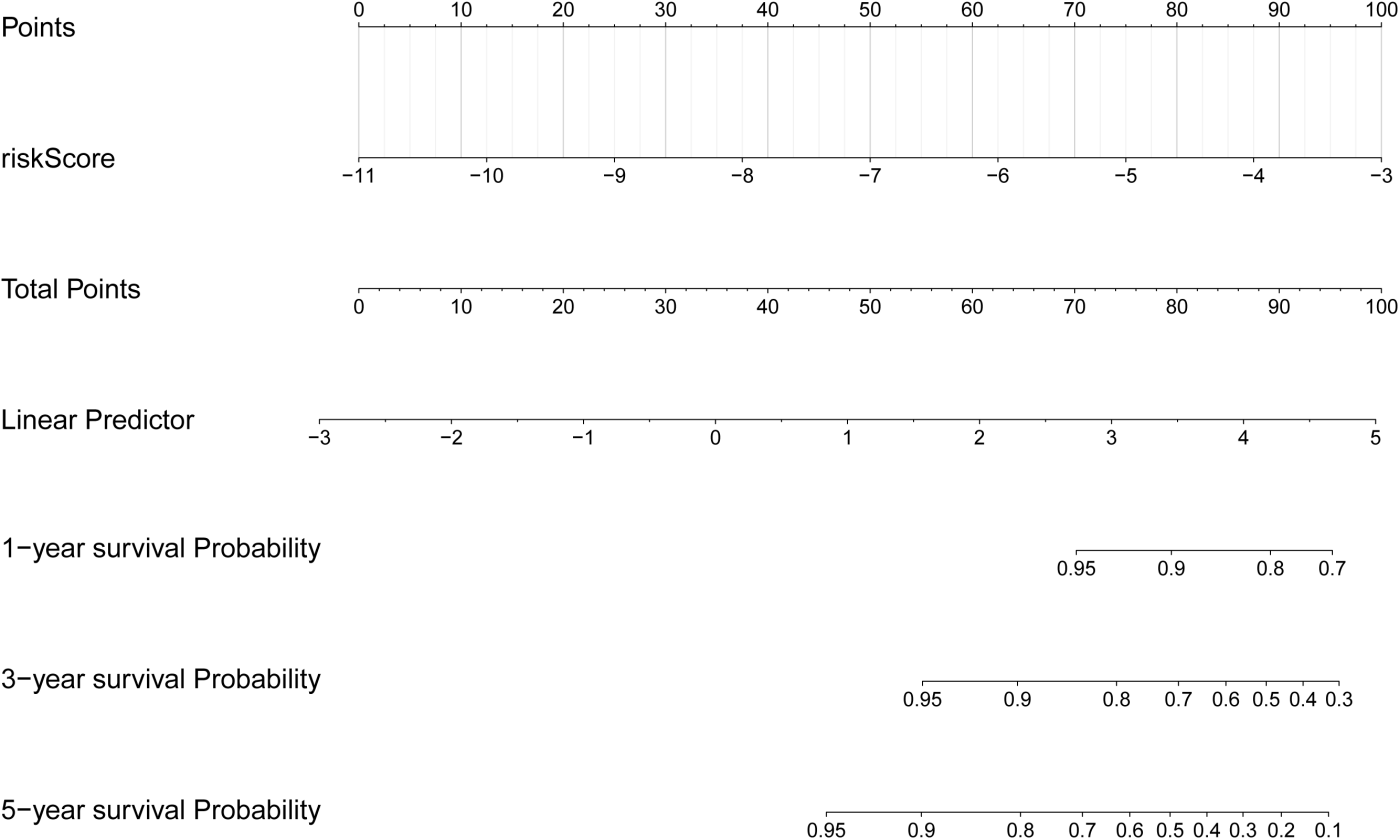
Creation of the nomogram based on the risk score.

Based on the clinical correlation analysis, the expression levels of CXCL8 exhibited significant variations among different subtypes of N stage (0/1) and T stage (1/2/3/4), while METTL7B demonstrated differential expression between the TNM stages (I/II/III/IV) and T stage (1/2/3/4), and TPD52L1 displayed alterations at M stage (0/1) (**Supplementary Figure S2A–E**, **Supplementary Table S5**). To demonstrate the clinical value of the risk score more directly, we constructed a nomogram model that mapped the risk score onto the total number axis to determine patients’ overall scores and subsequently derive their corresponding predicted survival probabilities (**Figure 5**). Meanwhile, the calibration curve of the nomogram demonstrated its excellent predictive capability (**Supplementary Figure S2F**).

### 3.5 Relevance of prognostic genes and immune environment

**Figure 6:**
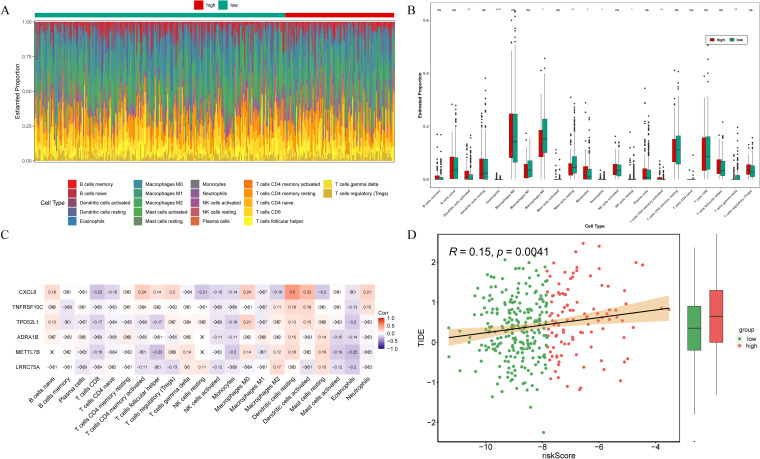
Immune infiltration analysis. **(A)** Estimated proportion of 22 immune cell types in the samples from high- and low-risk groups. **(B)** Comparison of the proportion of immunoinfiltrating cells in samples from high- and low-risk groups. ns, not significant; *p<0.05; **p<0.01; ****p<0.0001. **(C)** Heat map illustrating the correlation between prognostic genes and immune cells. **(D)** Differences of TIDE scores between high- and low-risk groups and correlation with risk score.

To investigate the correlation between risk signature and the immune microenvironment in THCA patients, an analysis of immunoinfiltration was conducted. **Figure 6A** exhibited the proportion of immune infiltration cells in the high- and low-risk groups. The high-risk group exhibited significantly elevated expression of activated dendritic cells, eosinophils, monocytes, neutrophils, and activated memory CD4 T cells among the total of 9 kind of differential immune cells. Conversely, the low-risk group demonstrated highly expressed M2 macrophages, resting mast cells, resting NK cells, and gamma delta T cells (**Figure 6B**). Correlation analysis revealed that CXCL8 displayed the strongest positive correlation with resting dendritic cells (cor = 0.5) (**Figure 6C**). What’s more, the TIDE scores presented significant disparities between the low- and high-risk groups, with the latter demonstrating a heightened likelihood of immune evasion (**Figure 6D**). Additionally, there was a positive correlation observed between the risk score and TIDE score (**Figure 6D**). Therefore, the risk signatures related to anoikis can be utilized for evaluating immune infiltration and immune evasion in patients with THCA.

### 3.6 Exploration of potential functions of prognostic genes

**Figure 7:**
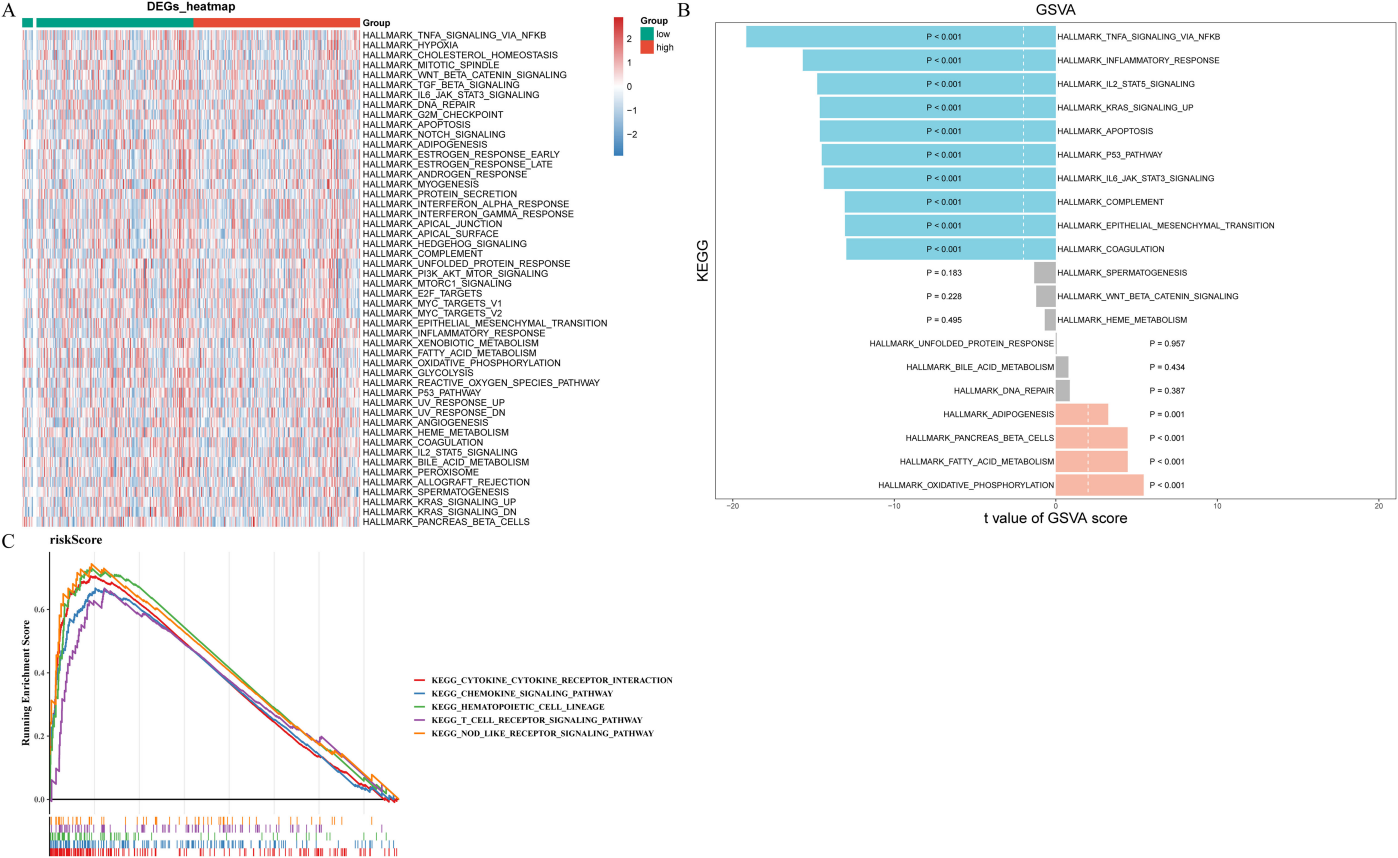
Functional enrichment analysis of prognostic genes and risk score. **(A)** Heat map of pathways exhibiting significant disparities between two risk groups in GSVA enrichment analysis. **(B)** The barplot of top20 pathways with significant differences. Blue represents inhibition, orange represents promotion, and gray represents not significant. **(C)** The results of GSEA enrichment analysis for risk score.

To determine the pathways for which prognostic gene enrichment differed between high- and low-risk groups, we conducted GSEA and GSVA enrichment analyses. **Figure 7A** displayed the pathways exhibiting significant disparities between two risk groups, highlighting the most prominent distinction where TNFA, IL2 STAT5, KRAS, P53, IL6 JAK STAT3 signaling pathways, inflammatory, and apoptosis were significantly inhibited while oxidative phosphorylation, fatty acid metabolism, and adipogenesis is significantly activated (**Figure 7B**). According to the results of GSEA, risk score was associated with chemokine, T cell receptor, and nod like receptor signaling pathways, cytokine receptor interaction, and hematopoietic cell lineage (**Figure 7C**).

### 3.7 Comparison of drug sensitivity between groups

**Figure 8:**
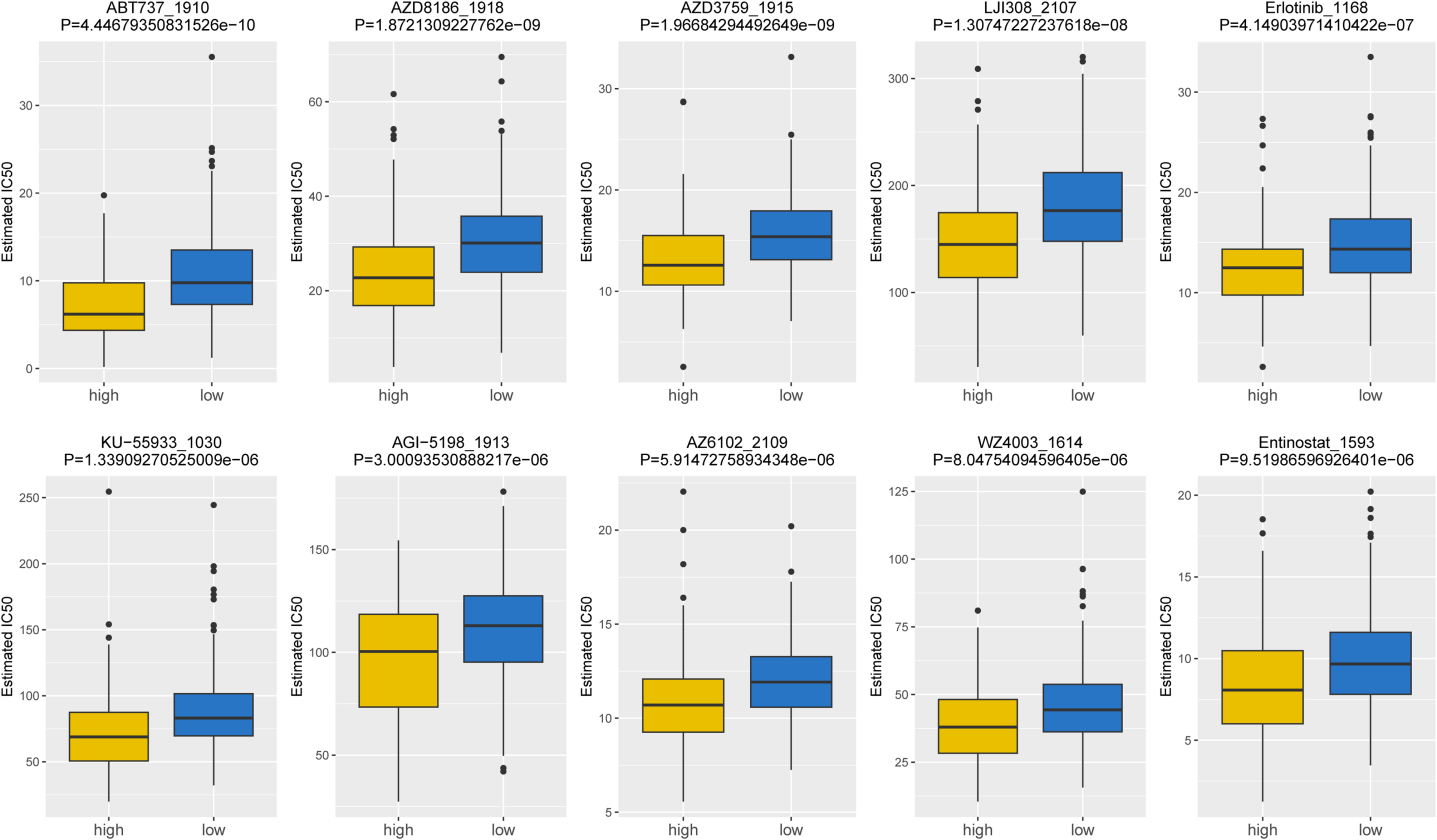
Top10 chemotherapeutic drugs exhibiting significant differences in IC50 values between high- and low-risk groups (top10).

Based on the data from GDSC database, the IC50 values of 107 drugs exhibited significant variations between two risk groups (high and low), implying potential divergent therapeutic effects in patients at different stages of tumor progression. For the purpose of demonstration, we presented the top10 drugs exhibiting significant differences in **Figure 8**, including ABT737_1910, AZD3759_1915, Erlotinib_1168, etc. In conclusion, our findings suggested that the risk score played a beneficial role in predicting drug effectiveness.

### 3.8 Causal effect of prognostic genes on THCA

**Figure 9:**
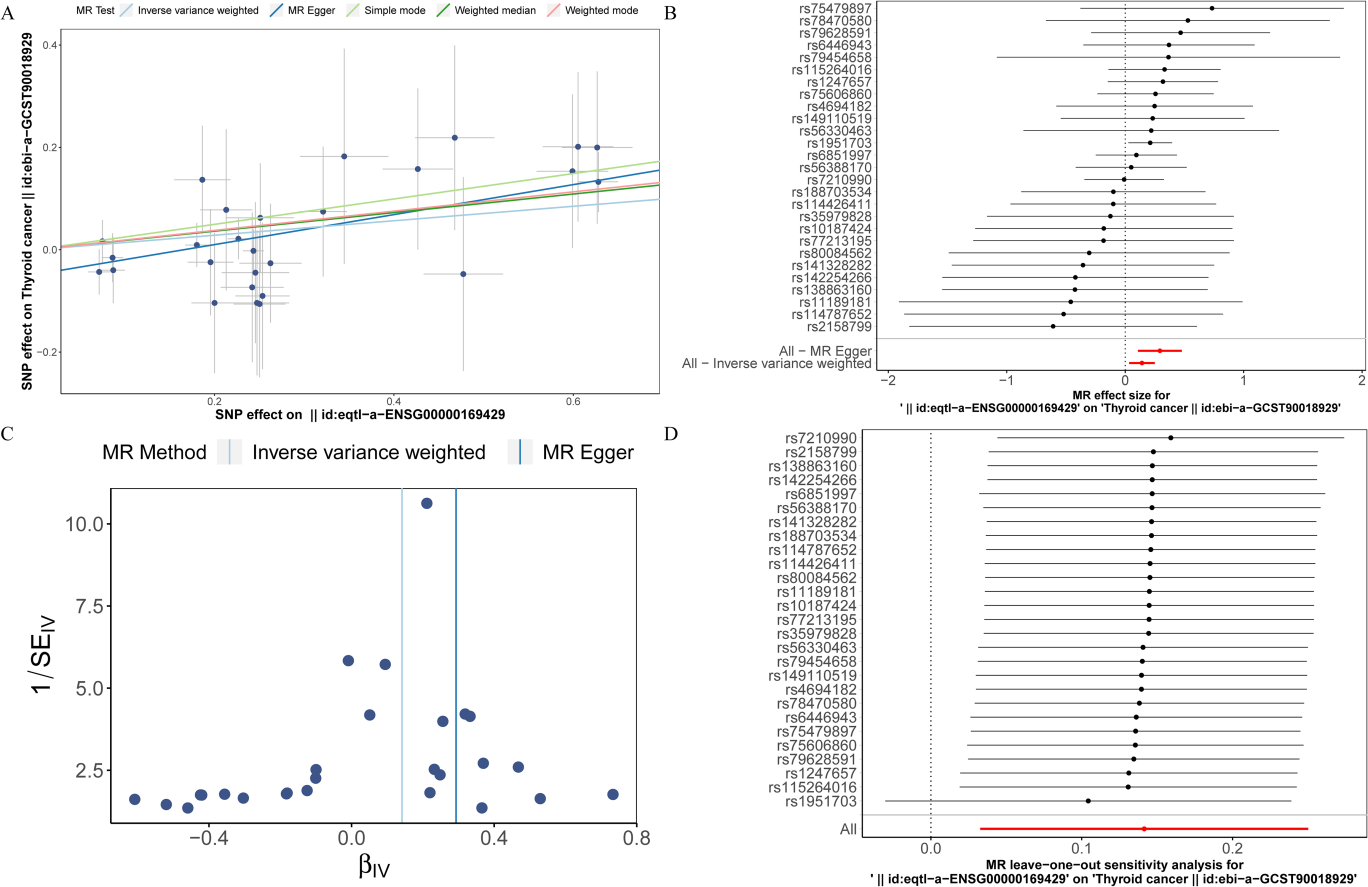
Mendelian randomization (MR) analysis between CXCL8 and THCA. **(A)** Scatter plot of the effect of single-nucleotide polymorphisms (SNPs) on CXCL8 (exposure) and THCA (outcome). **(B)** The forest map illustrates the impact of each SNP of CXCL8 on THCA. **(C)** MR-Egger regression funnel plot for CXCL8 on THCA. **(D)** MR leave-one-out sensitivity analysis for the effect of CXCL8 on THCA.

To investigate the causal relationship between prognostic genes and THCA, we conducted a two-sample MR analysis, utilizing prognostic genes as exposure factors and THCA as the outcome. Unfortunately, no GWAS IDs were found for ADRA1B, TPD52L1, and TNFRSF10C, hence they were excluded from MR analysis. According to the results from IVW method, only CXCL8 demonstrated a causal relationship with THCA and was identified as a risk factor for THCA [odds ratio (OR) = 1.1520, 95% confidence interval (CI): 1.0333-1.2843, p = 0.0108]. There was no significant causal association between LRRC75A or METTL7B and THCA (p > 0.05) (**Table 1**, **Figure 9A**). The Steiger direction check was confirmed as TRUE, indicating the presence of correct causation and the absence of reverse causation (**Supplementary Table S6**). The MR analysis included a total of 27 SNPs, all of which exhibited F value exceeding 10, indicating their suitability as strong IVs (**Table 2**). Meanwhile, the risk effects of the estimated exposure factors at each SNP site were combined to predict the diagnostic efficacy on THCA within the forest plot. As depicted in **Figure 9B**, IVW analysis revealed that the impact values of CXCL8 on THCA were all greater than 0. In order to assess the randomness of the analysis, a funnel plot was constructed by combining the β coefficient and standard error (SE) for each IVs (**Figure 9C**). The distribution of IVs in the plot appeared slightly uneven, which could be attributed to heterogeneity and potential bias resulting from a limited number of IVs. Consequently, a subsequent test for heterogeneity was conducted, yielding the p value greater than 0.05, indicating it had no impact on the overall findings (**Supplementary Table S7**). Moreover, the horizontal pleiotropy test conducted on CXCL8 indicated a p value exceeding 0.05, suggesting the absence of any significant horizontal pleiotropic effects (**Supplementary Table S8**). The absence of any confounding factors was found in this study, thereby further enhancing the robustness of our analysis. Additionally, there were no abnormal SNP results observed in relation to the leave-one-out analysis (**Figure 9D**). In summary, these findings suggested a significant causal relationship between CXCL8 and THCA, indicating that upregulation of CXCL8 expression was associated with an increased risk of THCA.

**Table 1:** Mendelian randomization (MR) analysis of prognostic genes on THCA.

Outcome	Exposure	Gene	Method	Number of SNPs	Beta	SE	P value	OR	or_lci95	or_uci95
ebi-a-GCST90018929 (Thyroid cancer)	eqtl-a- ENSG00000169429	CXCL8	MR Egger	27	0.2930	0.0945	0.0047	1.3405	1.1138	1.6133
			Weighted median		0.1814	0.0795	0.0226	1.1989	1.0258	1.4011
			Inverse variance weighted (fixed effects)		0.1415	0.0555	0.0108	1.1520	1.0333	1.2843
			Simple mode		0.2480	0.1269	0.0616	1.2814	0.9992	1.6434
			Weighted mode		0.1885	0.0803	0.0268	1.2074	1.0316	1.4131
	eqtl-a- ENSG00000181350	LRRC75A	MR Egger	10	-0.0691	0.3049	0.8263	0.9332	0.5133	1.6965
			Weighted median		-0.1283	0.1945	0.5094	0.8796	0.6008	1.2877
			Inverse variance weighted (fixed effects)		-0.2014	0.1527	0.1872	0.8175	0.6060	1.1029
			Simple mode		-0.1708	0.3114	0.5966	0.8430	0.4579	1.5519
			Weighted mode		-0.1050	0.2245	0.6512	0.9004	0.5799	1.3980
	eqtl-a- ENSG00000170439	METTL7B	MR Egger	6	0.4188	0.2482	0.1668	1.5202	0.9346	2.4727
			Weighted median		0.2058	0.1365	0.1317	1.2285	0.9401	1.6053
			Inverse variance weighted (fixed effects)		0.1749	0.1291	0.1756	1.1911	0.9248	1.5342
			Simple mode		-0.1276	0.2755	0.6627	0.8802	0.5130	1.5103
			Weighted mode		0.2749	0.1552	0.1367	1.3165	0.9711	1.7846

SNPs, single-nucleotide polymorphisms; SE, standard error; OR, odds ratio; CI, confidence interval.

**Table 2:** The list comprises SNPs of CXCL8.

beta	pval	samplesize	se	Chr	SNP	effect_ allele	other_ allele	eaf	r2	F
0.0863023	7.41E-13	25646	0.0120337	2	rs10187424	C	T	0.420816	0.002001505	51.42952458
-0.467891	3.66E-26	19193	0.0442212	4	rs79628591	C	T	0.0183072	0.005799087	111.9394266
-0.18639	2.70E-09	19726	0.0313339	4	rs75479897	A	G	0.037387	0.001790601	35.38117695
-0.241996	5.72E-12	3989	0.0351416	4	rs80084562	G	A	0.029456	0.011748349	47.3975084
0.251005	2.56E-11	20823	0.0376319	4	rs4694182	C	A	0.97441	0.002131976	44.48470741
-0.344598	2.68E-12	4750	0.0492755	4	rs78470580	A	G	0.0147564	0.010191102	48.88555231
-0.477385	3.75E-27	20197	0.0442355	4	rs188703534	A	G	0.0182892	0.005733392	116.4535276
-0.247371	4.35E-12	3757	0.03572	4	rs142254266	A	G	0.0284802	0.012604471	47.93397171
0.226612	4.54E-79	25646	0.0120364	4	rs6851997	G	A	0.392856	0.013633001	354.436724
-0.24557	6.63E-11	6617	0.0376139	4	rs77213195	G	A	0.0256185	0.006400357	42.61108602
-0.605328	2.22E-53	25143	0.0393574	4	rs115264016	G	A	0.0230215	0.009320619	236.5343275
-0.200047	8.07E-15	1926	0.0257586	4	rs114787652	T	A	0.0563344	0.030364884	60.25156896
0.627988	6.58E-179	24595	0.0220191	4	rs1951703	C	T	0.925346	0.032012992	813.3327223
-0.250272	5.17E-18	2101	0.0289345	4	rs138863160	C	T	0.0440267	0.034385114	74.74445002
-0.599117	8.28E-51	24449	0.0399627	4	rs75606860	T	C	0.022332	0.00910916	224.7388283
-0.213024	2.41E-13	1926	0.0290866	4	rs79454658	T	C	0.0436137	0.027094766	53.58212564
-0.426675	1.35E-27	24173	0.0391966	4	rs6446943	G	T	0.0234126	0.004878018	118.4845345
-0.253516	5.99E-17	2614	0.0303057	4	rs141328282	T	C	0.039977	0.026072528	69.92455281
-0.626673	4.33E-57	24954	0.039355	4	rs1247657	T	C	0.0229965	0.010058919	253.5404897
-0.26234	3.35E-14	20757	0.0345908	4	rs114426411	C	T	0.0304098	0.002763385	57.51299289
-0.0750399	4.75E-10	25531	0.0120507	5	rs56330463	C	T	0.582097	0.001516467	38.77268896
-0.321043	9.87E-34	19697	0.0265204	6	rs149110519	T	C	0.0526158	0.007384941	146.5285223
0.0713426	6.34E-09	25531	0.0122848	7	rs2158799	G	C	0.625845	0.00131923	33.72311334
0.180296	1.75E-43	25317	0.0130393	7	rs56388170	T	G	0.289189	0.007495213	191.1742164
-0.0870547	2.12E-11	25531	0.0129985	10	rs11189181	G	A	0.297492	0.001753751	44.85015488
-0.195535	2.18E-14	23798	0.0255963	12	rs35979828	T	C	0.0571059	0.002446192	58.35232007
0.243392	1.42E-95	25531	0.0117337	17	rs7210990	A	C	0.481977	0.016573593	430.2378514

### 3.9 Verification of prognostic genes expression by qPCR

**Figure 10:**
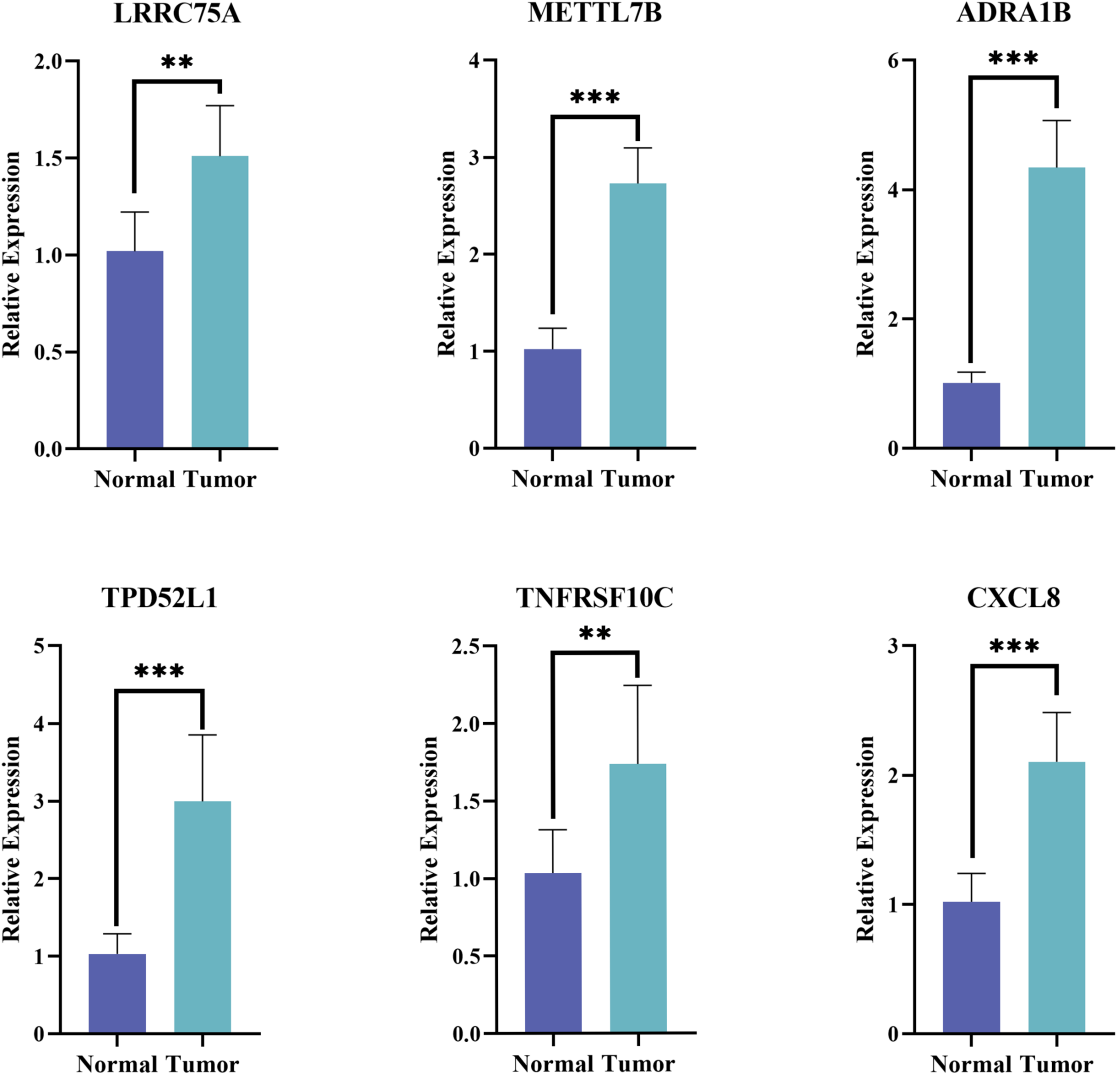
The results of qPCR for prognostic genes. **p<0.01; ***p<0.001.

The expression of the six prognostic genes was then verified in tumor and normal samples by qPCR. Consistent with the prediction, the results showed that the expression levels of the selected genes were remarkable elevated in THCA patients (p*<* 0.01) (**Figure 10**).

## 4 Discussion

The incidence of THCA is steadily increasing recent decades, making it the most prevalent endocrine cancer with relatively low mortality rates, partly due to overdiagnosis over world (21, 22). However, almost 10% of THCA exhibit recurrent or metastatic disease, the early appropriate treatment and accurate diagnosis of metastatic TCHA is still challenging (23, 24). When cells fail to attach to the ECM or adhere in inappropriate locations, they undergo a specific form of apoptosis known as anoikis. If the anoikis process is not properly executed, it may result in the rapid proliferation of cells in ectopic sites. This dysregulation of anoikis has become a hallmark of cancer cells, contributing to their metastasis to distant organs (25, 26). Metastasis, particularly to the lungs and bones, is the primary cause for worsen survival (27). Therefore, constructing risk signature associated with metastasis can offer valuable insights for disease management and early intervention for THCA.

In this study, we identified a total of 3,772 DEGs between normal and tumor samples from the TCGA-THCA dataset. After intersecting with ARGs, we obtained 570 DE-ARGs. Functional analysis revealed that these DE-ARGs primarily enriched in P53 signaling pathway, focal adhesion, transcriptional dysregulation, and proteoglycan pathways in cancer, highlighting the pivotal role of ARGs in tumorigenesis. Subsequently, utilizing univariate Cox regression, PH assumption test and LASSO analysis techniques, we further identified six prognostic genes: LRRC75A, METTL7B, ADRA1B, TPD52L1, TNFRSF10C, and CXCL8. The PCR results showed that all six prognostic genes were significantly overexpressed in THCA tissues. Notably, these findings were consistent with the differential expression analysis, with each gene exhibiting a log_2_FC greater than 1. These findings confirm our initial conclusions and underscore the importance of these genes in the context of THCA.

Leucine-rich repeat-containing protein 75A (LRRC75A) is a LRRC-rich protein belonging to the LRRC superfamily, yet its functionality remains largely unknown (28). However, based on scRNA-seq profiling, Miura T et al. have suggested that LRRC75A may positively regulate VEGF expression and serve as a potential biomarker for predicting BM-MSC angiogenesis under ischemic conditions (28). Notably, some effective therapeutic drugs for THCA act through the angiogenesis pathway involving VEGFR2, the receptor of VEGF (29). Several studies have demonstrated the high expression of Methyltransferase-like 7B (METTL7B) in THCA, which is consistent with our findings. It is suggested that METTL7B may promote TGF-β1-induced epithelial-mesenchymal transition (EMT), thereby enhancing the migration and invasion of THCA cells (30, 31). Adrenoceptor alpha 1B (ADRA1B) belongs to the adrenergic receptor alpha1 (ADRA1) subfamily, and adrenergic receptor antagonists have been reported to play a role in treating various cancers such as prostate and breast cancer, besides its significant upregulation in THCA samples (32–34). Therefore, we conclude that ADRA1B plays a crucial role in the occurrence and progression of THCA; however, further investigation is required to elucidate its underlying mechanism. Tumor protein D52-like 1 (TPD52L1) belongs to the TPD52 family; previous research has shown that TPD52 can act as a novel regulator of LKB1-AMPK pathway by negatively regulating AMPK activity and thus affecting cancer cell metabolism (35). As early as 2014, Andrade BM’s team proposed that AMPK regulated iodide and glucose uptake in normal thyroid cells and was highly activated in THCA (36, 37). However, immunohistochemical analysis revealed no activation of the AMPK pathway in adenoma tissue compared to non-tumor tissue from the same patient (36). Nevertheless, we still hypothesize that TPD52L1 may be involved in the development of THCA through its regulation of the AMPK pathway, and further investigation into its mechanism is urgently needed. Additionally, TNF receptor superfamily member 10C (TNFRSF10C) inhibits intracellular signaling pathways for apoptosis, and its high methylation is significantly associated with an increased risk in CRC patients (38). Although the precise role of TNFRSF10C in THAC remains unclear, further investigation is needed to explore the potential link between the significantly inhibited TNF-α/NF-κB signaling pathway identified in GSVA analysis and TNFRSF10C as one of the decoy receptors for TNF-associated apoptosis-inducing ligand (TRAIL). C-X-C motif chemokine ligand 8 (CXCL8 or IL-8) is a chemokine that exerts autocrine or paracrine regulation on tumor proliferation, invasion, and migration. It has been implicated in various cancers such as breast cancer, prostate cancer, lung cancer, and others (39). The relationship between CXCL8 and thyroid gland was confirmed as early as 1992. Experimental evidence has demonstrated that the presence of CXCL8 is essential for inducing EMT in THCA cells (40). Recent studies have further revealed higher levels of IL-6 and CXCL8 in THCA tissues compared to normal tissues, which aligns with our findings (41). Furthermore, GSEV result spot that tumor promotive signal such KRAS, EMT and IL-6/JAK/STAT3 are restricted under anoikis-related condition. Neoplastic diseases involve constant interactions, adaptations, and evolutions between tumor cells at different stages, involving surrounding normal cells, immune cells, integrity with sequential activation tumorigenesis signa-pathways (42, 43). During the early stages of tumor detachment from extracellular matrix environment, types are linked to promote progression (growth/infiltration into surrounding tissues) that could be summarized among T stage category. Subsequently, when overwhelming metastasis was aroused, the EMT-related and other pathways is initially activated by detachment from the extracellular matrix into circulation (lymph or blood circulation), triggering activation of anoikis-related signal-pathways. Our findings demonstrate that EMT and anoikis are two sequential and independent procedures promote tumorigenesis. This is also the main reason for further MR analysis after the construction of the prognostic signature contained anoikis-related genes.

The risk signature was constructed based on prognostic genes, and the THCA samples were stratified into high- and low-risk groups. The prognosis of patients in the low-risk group exhibited significantly better outcomes compared to those in the high-risk group. Utilizing the ROC curve analysis, we observed that the risk signature demonstrated a robust predictive ability for 1, 3, and 5-year survival probabilities in THCA patients. Furthermore, we developed a nomogram model incorporating risk scores to further elucidate the clinical significance of these risk signatures. In our clinical correlation analysis, we found that risk scores varied to different extents across distinct clinical characteristics. Consequently, can risk scores be employed to differentiate between subtypes of THCA patients? If feasible, this study would provide valuable insights for staging diagnosis and personalized treatment strategies for THCA patients; thus, warranting further investigation.

It is widely acknowledged that immunotherapy holds great promise as a treatment modality for various tumors. To identify personalized treatments for patients with different risk levels of THCA, we conducted an analysis on the disparities in the immune microenvironment between these two groups. Our immunoinfiltration analysis revealed significant discrepancies in the proportions of infiltration among nine types of immune cells, including M2 macrophages, NK cells, and diverse T cells. Shi ZY et al. (44), discovered a correlation between CD4 T cell count and clinical treatment efficacy, as well as adaptability and recovery potential in THCA patients; this correlation exhibited a negative trend. In our findings, we observed that the proportion of activated memory CD4 T cells were significantly higher in the high-risk group compared to the low-risk group. On the one hand, M2 macrophages have obvious immunosuppressive function to mediate immune escape and promote the proliferation and metastasis of THCA; on the other hand, it can also secrete VEGF and EGF to promote tumor microangiogenesis (45). Through the calculation and comparison of TIDE scores, we observed significant disparities between high- and low-risk groups, with samples from the former exhibiting a higher likelihood of immune escape. Moreover, there was a positive correlation between risk score and TIDE score. Consequently, we posit that aberrant infiltration of immune cells may foster the progression of THCA, thereby offering a theoretical foundation for exploring THCA immunotherapy.

However, our research has certain limitations. Firstly, the samples we analyzed were obtained from public databases with a small sample size and limited clinical information, which may restrict the extent to which our risk model can be researched and validated in terms of its clinical value. Secondly, although the expression of these six hub genes had been verified by clinical samples, further studies are needed to confirm the role of these prognostic genes in THCA. Moreover, additional investigation is urgent to uncover mechanisms of anoikis and its association with THCA.

## 5 Conclusion

In conclusion, we employed bioinformatics methods to screen six prognostic genes (LRRC75A, METTL7B, ADRA1B, TPD52L1, TNFRSF10C, and CXCL8) associated with anoikis for THCA. Utilizing these prognostic genes as a basis, we developed a risk signature to accurately predict the prognosis of THCA patients and made predictions regarding potential functions and targeted drugs. Significantly advancing the field of research on THCA prognostication is our novel integration of MR with transcriptome analysis in order to identify prognostic genes exhibiting significant causal relationships. Of particular interest is our discovery through MR analysis that there exists a substantial causal relationship between CXCL8 and THCA; indeed, CXCL8 emerges as a major risk factor for this disease. This finding not only provides a theoretical foundation for further exploration into the association between ARGs and THCA but also offers an innovative target for enhancing prognosis and facilitating precise and effective treatment strategies for individuals afflicted by this condition.

## Data Availability

The original contributions presented in the study are included in the article/**Supplementary Material**. Further inquiries can be directed to the corresponding authors.

## References

[1] Boucai L Zafereo M Cabanillas ME. Thyroid cancer: A review. Jama. (2024) 331:425–35. doi: 10.1001/jama.2023.26348 38319329

[2] Nabhan F Dedhia PH Ringel MD. Thyroid cancer, recent advances in diagnosis and therapy. Int J Cancer. (2021) 149:984–92. doi: 10.1002/ijc.33690 34013533

[3] Li J Li Z Zhao P. Diagnosis and prognosis of thyroid cancer by immune-related genes. Am J Clin Oncol. (2024) 47:1–10. doi: 10.1097/coc.0000000000001048 37779238

[4] Taddei ML Giannoni E Fiaschi T Chiarugi P. Anoikis: an emerging hallmark in health and diseases. J Pathol. (2012) 226:380–93. doi: 10.1002/path.3000 21953325

[5] Lee JJ Hsu YC Li YS Cheng SP. Galectin-3 inhibitors suppress anoikis resistance and invasive capacity in thyroid cancer cells. Int J Endocrinol. (2021) 2021:5583491. doi: 10.1155/2021/5583491 34035807 PMC8124007

[6] Jensen K Patel A Klubo-Gwiezdzinska J Bauer A Vasko V. Inhibition of gap junction transfer sensitizes thyroid cancer cells to anoikis. Endocr Relat Cancer. (2011) 18:613–26. doi: 10.1530/erc-10-0289 21813730

[7] Tang M Luo W Zhou Y Zhang Z Jiang Z. Anoikis-related gene CDKN2A predicts prognosis and immune response and mediates proliferation and migration in thyroid carcinoma. Transl Oncol. (2024) 40:101873. doi: 10.1016/j.tranon.2023.101873 38141377 PMC10788268

[8] Pan Y Zhu X Wang K Chen Y. MicroRNA-363-3p suppresses anoikis resistance in human papillary thyroid carcinoma via targeting integrin alpha 6. Acta Biochim Biophys Sin (Shanghai). (2019) 51:807–13. doi: 10.1093/abbs/gmz066 31257410

[9] Diao X Guo C Li S. Identification of a novel anoikis-related gene signature to predict prognosis and tumor microenvironment in lung adenocarcinoma. Thorac Cancer. (2023) 14:320–30. doi: 10.1111/1759-7714.14766 PMC9870742 36507553

[10] Love MI Huber W Anders S. Moderated estimation of fold change and dispersion for RNA-seq data with DESeq2. Genome Biol. (2014) 15:550. doi: 10.1186/s13059-014-0550-8 25516281 PMC4302049

[11] Hänzelmann S Castelo R Guinney J. GSVA: gene set variation analysis for microarray and RNA-seq data. BMC Bioinf. (2013) 14:7. doi: 10.1186/1471-2105-14-7 PMC3618321 23323831

[12] Langfelder P Horvath S. WGCNA: an R package for weighted correlation network analysis. BMC Bioinf. (2008) 9:559. doi: 10.1186/1471-2105-9-559 PMC2631488 19114008

[13] Yu G Wang LG Han Y He QY. clusterProfiler: an R package for comparing biological themes among gene clusters. Omics. (2012) 16:284–7. doi: 10.1089/omi.2011.0118 PMC3339379 22455463

[14] Shi Y Wang Y Dong H Niu K Zhang W Feng K. Crosstalk of ferroptosis regulators and tumor immunity in pancreatic adenocarcinoma: novel perspective to mRNA vaccines and personalized immunotherapy. Apoptosis. (2023) 28:1423–35. doi: 10.1007/s10495-023-01868-8 PMC10425492 37369808

[15] Engebretsen S Bohlin J. Statistical predictions with glmnet. Clin Epigenetics. (2019) 11:123. doi: 10.1186/s13148-019-0730-1 31443682 PMC6708235

[16] Zhang S Sun L Cai D Liu G Jiang D Yin J. Development and validation of PET/CT-based nomogram for preoperative prediction of lymph node status in esophageal squamous cell carcinoma. Ann Surg Oncol. (2023) 30:7452–60. doi: 10.1245/s10434-023-13694-y 37355519

[17] Liu M Gao Y Yuan Y Liu X Wang Y Li L. A comprehensive study of clinicopathological and genetic features of neuronal intranuclear inclusion disease. Neurol Sci. (2023) 44:3545–56. doi: 10.1007/s10072-023-06845-2 37184590

[18] Grieder S Steiner MD. Algorithmic jingle jungle: A comparison of implementations of principal axis factoring and promax rotation in R and SPSS. Behav Res Methods. (2022) 54:54–74. doi: 10.3758/s13428-021-01581-x 34100201 PMC8863761

[19] Maeser D Gruener RF Huang RS. oncoPredict: an R package for predicting in *vivo* or cancer patient drug response and biomarkers from cell line screening data. Brief Bioinform. (2021) 22:1–7. doi: 10.1093/bib/bbab260 34260682 PMC8574972

[20] Huang Y Lin W Zheng X. Causal association between 637 human metabolites and ovarian cancer: a mendelian randomization study. BMC Genomics. (2024) 25:97. doi: 10.1186/s12864-024-09997-3 38262941 PMC10804684

[21] Liu Y Wang J Hu X Pan Z Xu T Xu J. Radioiodine therapy in advanced differentiated thyroid cancer: Resistance and overcoming strategy. Drug Resist Updat. (2023) 68:100939. doi: 10.1016/j.drup.2023.100939 36806005

[22] Li M Maso LD Vaccarella S. Global trends in thyroid cancer incidence and the impact of overdiagnosis. Lancet Diabetes Endocrinol. (2020) 8:468–70. doi: 10.1016/s2213-8587(20)30115-7 32445733

[23] Fugazzola L Elisei R Fuhrer D Jarzab B Leboulleux S Newbold K. 2019 European thyroid association guidelines for the treatment and follow-Up of advanced radioiodine-Refractory thyroid cancer. Eur Thyroid J. (2019) 8:227–45. doi: 10.1159/000502229 PMC6873012 31768334

[24] Shobab L Gomes-Lima C Zeymo A Feldman R Jonklaas J Wartofsky L. Clinical, pathological, and molecular profiling of radioactive iodine refractory differentiated thyroid cancer. Thyroid. (2019) 29:1262–8. doi: 10.1089/thy.2019.0075 31319763

[25] Wang J Luo Z Lin L Sui X Yu L Xu C. Anoikis-associated lung cancer metastasis: mechanisms and therapies. Cancers (Basel). (2022) 14:4791. doi: 10.3390/cancers14194791 36230714 PMC9564242

[26] Zhao K Wang Z Hackert T Pitzer C Zöller M. Tspan8 and Tspan8/CD151 knockout mice unravel the contribution of tumor and host exosomes to tumor progression. J Exp Clin Cancer Res. (2018) 37:312. doi: 10.1186/s13046-018-0961-6 30541597 PMC6292129

[27] Toraih EA Hussein MH Zerfaoui M Attia AS Marzouk Ellythy A Mostafa A. Site-specific metastasis and survival in papillary thyroid cancer: the importance of brain and multi-organ disease. Cancers (Basel). (2021) 13:1625. doi: 10.3390/cancers13071625 33915699 PMC8037301

[28] Miura T Kouno T Takano M Kuroda T Yamamoto Y Kusakawa S. Single-cell RNA-seq reveals LRRC75A-expressing cell population involved in VEGF secretion of multipotent mesenchymal stromal/stem cells under ischemia. Stem Cells Transl Med. (2023) 12:379–90. doi: 10.1093/stcltm/szad029 PMC10267575 37263619

[29] Enokida T Tahara M. Management of VEGFR-targeted TKI for thyroid cancer. Cancers (Basel). (2021) 13:5536. doi: 10.3390/cancers13215536 34771698 PMC8583039

[30] Ye D Jiang Y Sun Y Li Y Cai Y Wang Q. METTL7B promotes migration and invasion in thyroid cancer through epithelial-mesenchymal transition. J Mol Endocrinol. (2019) 63:51–61. doi: 10.1530/jme-18-0261 31121562

[31] Ali J Liu W Duan W Liu C Song J Ali S. METTL7B (methyltransferase-like 7B) identification as a novel biomarker for lung adenocarcinoma. Ann Transl Med. (2020) 8:1130. doi: 10.21037/atm-20-4574 33240979 PMC7576055

[32] Freudenberger RS Kim J Tawfik I Sonnenberg FA. Optimal medical therapy is superior to transplantation for the treatment of class I, II, and III heart failure: a decision analytic approach. Circulation. (2006) 114:I62–66. doi: 10.1161/circulationaha.105.001412 16820647

[33] Harris AM Warner BW Wilson JM Becker A Rowland RG Conner W. Effect of alpha1-adrenoceptor antagonist exposure on prostate cancer incidence: an observational cohort study. J Urol. (2007) 178:2176–80. doi: 10.1016/j.juro.2007.06.043 PMC2084470 17870114

[34] Zhong LK Deng XY Shen F Cai WS Feng JH Gan XX. Identification of a 3-gene prognostic index for papillary thyroid carcinoma. Front Mol Biosci. (2022) 9:807931. doi: 10.3389/fmolb.2022.807931 35372518 PMC8966665

[35] Chen Y Peng C Tan W Yu J Zayas J Peng Y. Tumor protein D52 (TPD52) affects cancer cell metabolism by negatively regulating AMPK. Cancer Med. (2023) 12:488–99. doi: 10.1002/cam4.4911 PMC9844640 35666017

[36] Andrade BM de Carvalho DP. Perspectives of the AMP-activated kinase (AMPK) signalling pathway in thyroid cancer. Biosci Rep. (2014) 34:e00105. doi: 10.1042/bsr20130134 27919039 PMC3986867

[37] Vidal AP Andrade BM Vaisman F Cazarin J Pinto LF Breitenbach MM. AMP-activated protein kinase signaling is upregulated in papillary thyroid cancer. Eur J Endocrinol. (2013) 169:521–8. doi: 10.1530/eje-13-0284 23904275

[38] Zhou C Pan R Hu H Li B Dai J Ying X. TNFRSF10C methylation is a new epigenetic biomarker for colorectal cancer. PeerJ. (2018) 6:e5336. doi: 10.7717/peerj.5336 30225159 PMC6139245

[39] Liu Q Li A Tian Y Wu JD Liu Y Li T. The CXCL8-CXCR1/2 pathways in cancer. Cytokine Growth Factor Rev. (2016) 31:61–71. doi: 10.1016/j.cytogfr.2016.08.002 27578214 PMC6142815

[40] Rotondi M Coperchini F Latrofa F Chiovato L. Role of chemokines in thyroid cancer microenvironment: is CXCL8 the main player? Front Endocrinol (Lausanne). (2018) 9:314. doi: 10.3389/fendo.2018.00314 29977225 PMC6021500

[41] Zhang L Xu S Cheng X Wu J Wang Y Gao W. Inflammatory tumor microenvironment of thyroid cancer promotes cellular dedifferentiation and silencing of iodide-handling genes expression. Pathol Res Pract. (2023) 246:154495. doi: 10.1016/j.prp.2023.154495 37172523

[42] Tedja R Alvero AB Fox A Cardenas C Pitruzzello M Chehade H. Generation of stable epithelial-mesenchymal hybrid cancer cells with tumorigenic potential. Cancers (Basel). (2023) 15:684. doi: 10.3390/cancers15030684 36765641 PMC9913490

[43] Wang X Eichhorn PJA Thiery JP. TGF-β, EMT, and resistance to anti-cancer treatment. Semin Cancer Biol. (2023) 97:1–11. doi: 10.1016/j.semcancer.2023.10.004 37944215

[44] Shi ZY Zhang SX Li CH Fan D Xue Y Cheng ZH. Differential distribution and prognostic value of CD4(+) T cell subsets before and after radioactive iodine therapy in differentiated thyroid cancer with varied curative outcomes. Front Immunol. (2022) 13:966550. doi: 10.3389/fimmu.2022.966550 36091039 PMC9459039

[45] Liu Q Sun W Zhang H. Roles and new insights of macrophages in the tumor microenvironment of thyroid cancer. Front Pharmacol. (2022) 13:875384. doi: 10.3389/fphar.2022.875384 35479325 PMC9035491

