# Supplementary material for: Investigation of risk signatures associated with anoikis in thyroid cancer through integrated transcriptome and Mendelian randomization analysis
Source: Front Endocrinol (Lausanne). 2024 Nov 6;15:1458956. doi: 10.3389/fendo.2024.1458956 (PMC11576184; doi:10.3389/fendo.2024.1458956)
Supplement: Supplementary file 2 [file Presentation1.pdf]

Supplementary Figures

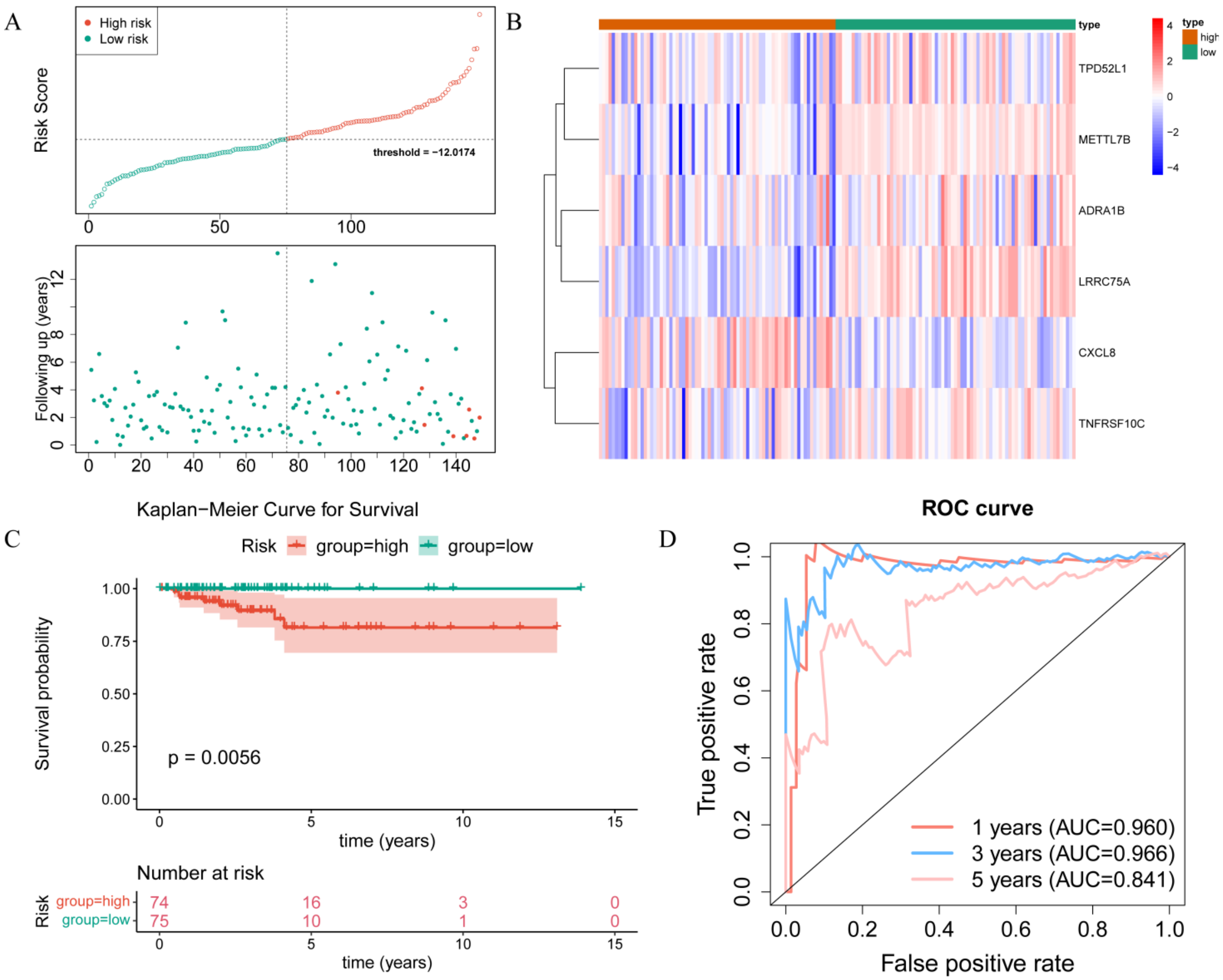

Supplementary Figure 1. Validation of the risk signature in the validation set. (A) Risk score distribution, patient survival time, and status. (B) The heat map illustrates the expression of prognostic genes. (C) K-M survival curves for high- and low-risk groups. (D) ROC curves showing the predictive efficiency of risk scores.

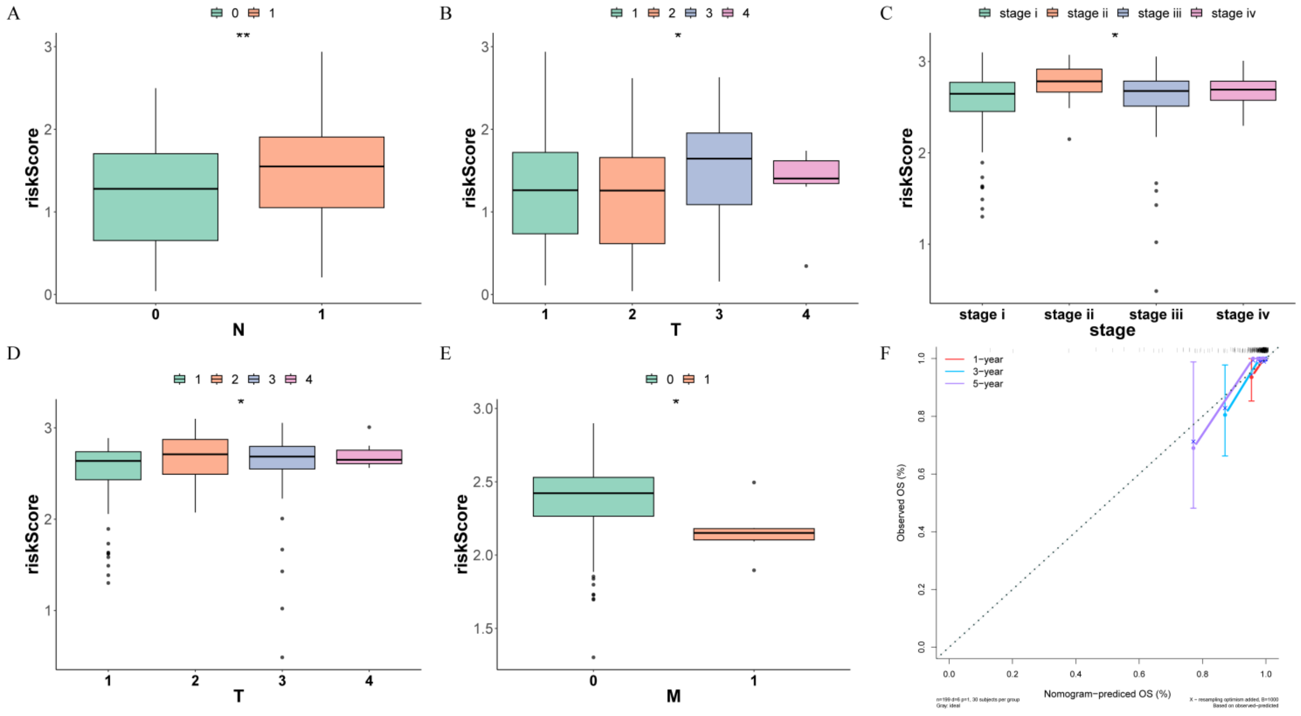

Supplementary Figure 2. Correlation analysis of risk score and clinical features. The discrepancies of risk score among different subtypes of N stage (A), T stage (B), TNM stage (C), T stage (D), and M stage (E). *p<0.05; **p<0.01. (F) The calibration curve of the nomogram.
